# 18. Global Surveillance of *Clostridioides difficile* Demonstrates High Prevalence in Non-Healthcare Settings

**DOI:** 10.1093/ofid/ofab466.018

**Published:** 2021-12-04

**Authors:** Jinhee Jo, Anne J Gonzales-Luna, Kevin W Garey

**Affiliations:** 1 University of Houston, Houston, Texas; 2 University of Houston College of Pharmacy, Houston, Texas

## Abstract

**Background:**

*Clostridioides difficile* is a Gram-positive, spore-forming, toxin-producing organism that is the leading cause of healthcare-associated infections. However, past studies have isolated *C. difficile* spores from the community, suggesting an environmental reservoir that may play a role in transmission. This study aimed to examine the prevalence and strain types of *C. difficile* isolated from the United States (US) and internationally.

**Methods:**

From 2014 to 2017, environmental swabs were collected from public areas, healthcare settings, and shoe soles. Samples were considered positive for *C. difficile* following growth on CCFA plates and confirmatory PCR testing for toxin genes and fluorescent PCR ribotyping (RT). The rate of *C. difficile* positivity and associated RT distribution were compared between settings, including shoe soles which were investigated for their potential role in environmental transmission.

**Results:**

A total of 11,986 unique isolates were obtained primarily from the US (n=11,002; 92%) in addition to 11 other countries including Taiwan (n=200) and India (n=187). Samples were categorized as being from outdoor environments (n=2,992), private residences (n=2,772), shoe soles (n=1,420), public buildings (n=1,104) or acute care settings (n=3,698). Worldwide *C. difficile* sample positivity was 26% and was similar between US and non-US sampling sites. In the US, private residences (26.2%) and outdoor environments (24.1%) had the highest positivity rate compared to public buildings (17.2%). In a Texas sub-analysis (n=8,571), positivity rates were highest from outdoor samples (27%) and were similar between private residences (24%) and healthcare buildings (24%). The most prevalent RTs overall were F014-020 (16.4%), F106 (14.9%), and FP310 (11%). Shoe soles had the highest positivity rate (45%) with similar RT distribution between shoe soles and environmental samples.

**Conclusion:**

Using a worldwide sample, 26% of environmental samples tested positive for toxigenic *C. difficile* strains from healthcare and non-healthcare sites. Community stewardship efforts will be needed to reduce the risk of CDI in vulnerable patients. Shoe sole sampling may be an ideal surveillance tool to test for emerging epidemic strains.

**Disclosures:**

**Kevin W. Garey, Pharm.D., M.S., FASHP**, **Summit Therapeutics** (Research Grant or Support)

